# Complete Reaction Phenotyping of Propranolol and 4-Hydroxypropranolol with the 19 Enzymes of the Human UGT1 and UGT2 Families

**DOI:** 10.3390/ijms23137476

**Published:** 2022-07-05

**Authors:** Fan Yang, Sijie Liu, Gerhard Wolber, Matthias Bureik, Maria Kristina Parr

**Affiliations:** 1 Pharmaceutical and Medicinal Chemistry (Pharmaceutical Analyses), Institute of Pharmacy, Freie Universität Berlin, 14195 Berlin, Germany; yangfano129@zedat.fu-berlin.de; 2 Pharmaceutical and Medicinal Chemistry (Computer-Aided Drug Design), Institute of Pharmacy, Freie Universität Berlin, 14195 Berlin, Germany; sijie.liu@fu-berlin.de (S.L.); gerhard.wolber@fu-berlin.de (G.W.); 3 School of Pharmaceutical Science and Technology, Tianjin University, Tianjin 300072, China; matthias@tju.edu.cn

**Keywords:** UGTs, propranolol glucuronide, 4-hydroxypropranolol glucuronide, in vitro metabolism, stereoselectivity, aromatic and aliphatic-linked glucuronidation

## Abstract

Propranolol is a competitive non-selective beta-receptor antagonist that is available on the market as a racemic mixture. In the present study, glucuronidation of propranolol and its equipotent phase I metabolite 4-hydroxypropranolol by all 19 members of the human UGT1 and UGT2 families was monitored. UGT1A7, UGT1A9, UGT1A10 and UGT2A1 were found to glucuronidate propranolol, with UGT1A7, UGT1A9 and UGT2A1 mainly acting on (*S*)-propranolol, while UGT1A10 displays the opposite stereoselectivity. UGT1A7, UGT1A9 and UGT2A1 were also found to glucuronidate 4-hydroxypropranolol. In contrast to propranolol, 4-hydroxypropranolol was found to be glucuronidated by UGT1A8 but not by UGT1A10. Additional biotransformations with 4-methoxypropanolol demonstrated different regioselectivities of these UGTs with respect to the aliphatic and aromatic hydroxy groups of the substrate. Modeling and molecular docking studies were performed to explain the stereoselective glucuronidation of the substrates under study.

## 1. Introduction

UDP-glucuronosyltransferases (UGTs) are a superfamily of enzymes that catalyze the glucuronidation of a variety of endobiotics and xenobiotics. These enzymes transfer the sugar moiety from the cofactor UDP-glucuronic acid (UDPGA) to a nucleophilic group (such as carbonyl, carboxyl, sulfuryl, hydroxy or amine groups [1,2]) of the substrate, forming β-d-glucuronides [3] that are more water-soluble than the substrates and, therefore, readily excreted via urine or bile [4]. Arguably, UGTs are the most important enzymes in human phase II drug metabolism and have a diverse range of substrates, including bilirubin, sex steroids, thyroid hormones, bile acids and fat-soluble vitamins [5,6]. There are 22 UGTs in humans, which belong to the four families UGT1, UGT2, UGT3 and UGT8, respectively [7]. Among these, the 19 members of the families UGT1 and UGT2 appear to be more important for drug metabolism than the remaining three enzymes [8].

In addition to studying human UGTs from natural sources such as liver cells or human liver microsomes (HLMs), recombinant expression of human UGTs may be performed in cell culture or microbial systems [1,9,10,11]. We have repeatedly demonstrated that permeabilized fission yeast cells (enzyme bags) that recombinantly express human UGTs are a useful tool for the small-scale production of glucuronide metabolites [12,13,14].

Propranolol is a typical competitive non-selective beta-receptor antagonist and is commonly used to treat various cardiovascular disorders such as arrhythmia, hypertension and angina pectoris [15,16]. Because of its competitive effect with catecholamines, it is also used to treat anxiety disorder in some cases [17]. Propranolol is normally administered as racemic mixture [18,19], with (*S*)-propranolol being nearly 100 times more potent than its enantiomer. Diverse metabolic pathways exist for propranolol [20,21,22], and glucuronidation by UGTs is one of the most important ones. Because of the chiral structure of propranolol, UGT-catalyzed-glucuronidation of propranolol shows stereoselectivity [23,24,25]. Several publications showed that the concentration of (*R*)-propranolol glucuronide was lower than that of (*S*)-propranolol glucuronide in both plasma and urine upon intake of propranolol racemate [24,26]. In a study performed with recombinantly expressed enzymes of the human UGT1A and UGT2B subfamilies, UGT1A9 and UGT1A10 were reported to display opposite stereoselectivity in propranolol glucuronidation [23]. However, propranolol glucuronidated by the UGT2A subfamily has not yet been investigated.

4-Hydroxypropranolol is the major phase I metabolite of propranolol [25,27,28]. It has a longer half-life (5 to 8 h) than the parent compound (3 to 4 h) and is reported to be equipotent to propranolol with respect to beta-blocker activity [15,29]. Together with sulfation, glucuronidation is an important elimination pathway of 4-hydroxypropranolol [30]. In contrast to propranolol, it has two potential conjugation sites, the aliphatic hydroxy group on the side chain and the aromatic hydroxy group on the naphthol ring.

The aim of this study was to perform a complete reaction phenotyping of propranolol and 4-hydroxypropranolol with the 19 human members of the UGT1 and UGT2 families. For this purpose, a method was established for the analysis of intact glucuronides by LC-MS. The stereoselectivity of propranolol glucuronidation, as well as the stereo- and regioselectivities of 4-hydroxypropranolol glucuronidation, were also investigated. Finally, the observed catalytic activities were rationalized through homology modeling and molecular docking experiments. 

## 2. Results

### 2.1. Glucuronidation of Racemic Propranolol and Enantiopure (R)-Propranolol

**Figure 1:**
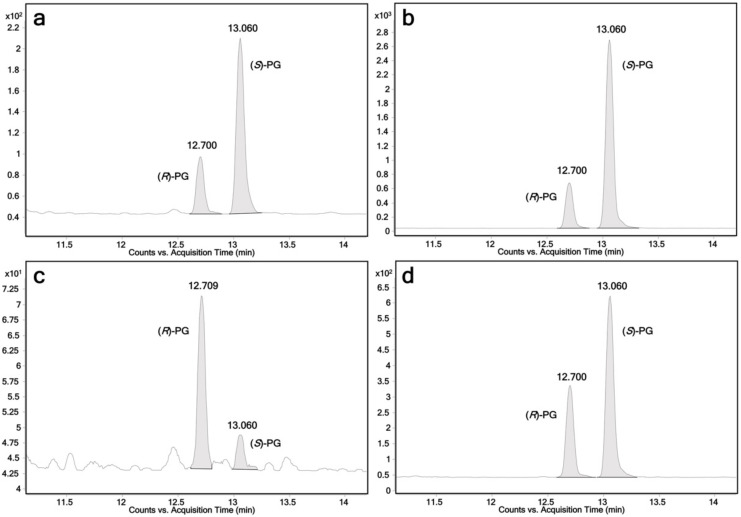
Chromatograms of (*R*)-propranolol glucuronide and (*S*)-propranolol glucuronide obtained from individual UGT reactions using racemic propranolol as the substrate. Ion transition *m*/*z* 436→116 is monitored; (*R*)-PG: (*R*)-propranolol glucuronide, retention time 12.7 min; (*S*)-PG: (*S*)-propranolol glucuronide, retention time 13.1 min; (**a**) UGT1A7; (**b**) UGT1A9; (**c**) UGT1A10; (**d**) UGT2A1.

**Figure 2:**
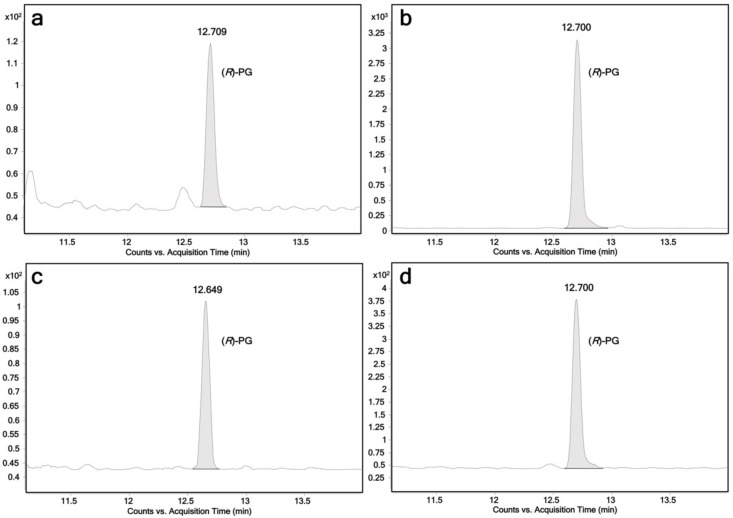
Chromatograms of (*R*)-propranolol glucuronide obtained from individual UGT reactions using (*R*)-propranolol as the substrate. Ion transition *m*/*z* 436→116 is monitored; (*R*)-PG: (*R*)-propranolol glucuronide, retention time 12.7 min; (**a**) UGT1A7; (**b**) UGT1A9; (**c**) UGT1A10; (**d**) UGT2A1.

In this study, the activity of all human members of the UGT1 and UGT2 families towards propranolol was tested. The enzymes were recombinantly expressed using fission yeast *Schizosaccharomyces pombe*. All fission yeast strains used in this study have been described previously [1,31,32]. Biotransformations using permeabilized fission yeast cells (enzyme bags) were performed using a protocol similar to those previously reported [12]. Samples analysis was performed by LC-MS/MS. Among the 19 UGTs, UGT1A7, UGT1A9, UGT1A10 and UGT2A1 were found to glucuronidate racemic propranolol. Due to the conjugation with the enantiopure β-d-glucuronic acid, two glucuronide diastereomers were detected in those four incubation samples (Figure 1a–d). In addition, (*R*)-propranolol was used as a substrate to assign the diastereomers. LC-MS chromatograms are shown in Figure 2. As expected, (*R*)-propranolol gave only one glucuronide product (retention time (RT) = 12.70 min). Thus, the other diastereomer (RT = 13.06 min) was assigned to (*S*)-propranolol glucuronide. An overview of the results on the activity of respective UGTs with different substrates is given in Table 1.

**Figure 3:**
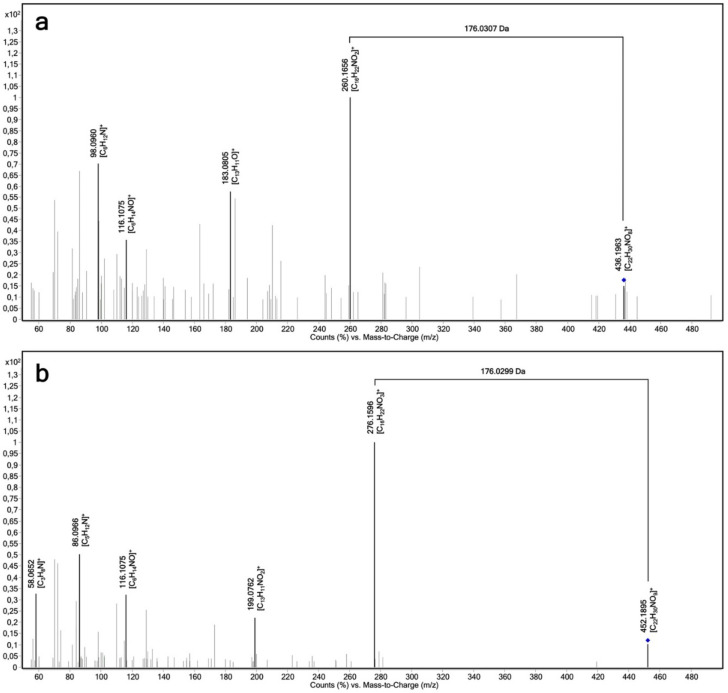
Product ion spectrum (LC-QTOF-MS) of propranolol glucuronide and 4-hydroxypropranolol glucuronide obtained by UGT-dependent biotransformations. (**a**) Propranolol glucuronide, C_22_H_29_NO_8_, [M+H]^+^ theor. = 436.1971, [M+H]^+^ exp. = 436.1963, ∆*m*/*z* = 0.3 ppm; (**b**) 4-hydroxypropranolol glucuronide, C_22_H_29_NO_9_, [M+H]^+^ theor. = 452.1895, [M+H]^+^ exp. = 452.1921, ∆*m*/*z* = 0.1 ppm.

The relative biocatalytic yields of (*R*)-propranolol glucuronide are 31.5%, 23.9% and 48.9% of (*S*)-propranolol glucuronide for UGT1A7, UGT1A9 and UGT2A1, respectively. This indicates that (*S*)-propranolol is the preferred substrate for these three enzymes. In contrast, the relative biocatalytic yield of (*S*)-propranolol glucuronide is 21.2% of (*R*)-propranolol glucuronide for UGT1A10, suggesting that UGT1A10 prefers this enantiomer. Tandem MS (MS^2^) analysis on a quadrupole time of flight (QTOF) instrument was performed for the additional characterization of propranolol glucuronide by high-resolution accurate mass spectrometry. A product ion spectrum is shown in Figure 3a. The loss of 176.0307 Da indicates the loss of the glucuronic acid moiety.

### 2.2. Glucuronidation Assay of Racemic 4-Hydroxypropranolol 

**Figure 4:**
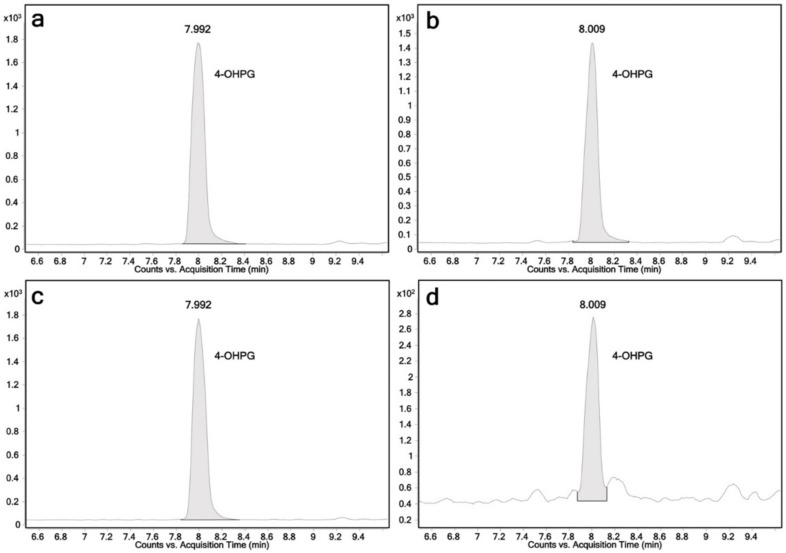
Chromatograms of 4-hydroxypropranolol glucuronide obtained from individual UGT reactions monitoring the ion transition *m*/*z* 452→116; 4-OHPG: 4-hydroxypropranolol glucuronide, retention time 8.0 min; (**a**) UGT1A7; (**b**) UGT1A8; (**c**) UGT1A9; (**d**) UGT2A1.

Biotransformation reactions with 4-hydroxypropranolol were performed using the same protocol as for the propranolol biotransformations. UGT1A7, UGT1A8, UGT1A9 and UGT2A1 were found to produce 4-hydroxypropranolol glucuronides. LC-MS chromatograms are shown in Figure 4. Only one glucuronide peak was detected (RT = 7.9 min). This may be explained by the limited separation capacity of the applied method for the 4-hydroxypropranolol isomers. MS^2^ analysis was performed for further characterization of 4-hydroxypropranolol glucuronide. The accurate mass product ion spectrum is shown in Figure 3b. As known for glucuronides, the dominant loss of the glucuronic acid moiety (loss of 176.0299 Da) hampers the final isomer assignment by fragmentation analysis.

### 2.3. Determination of the Regioselectivity of 4-Hydroxypropranolol Glucuronidation

As mentioned above, 4-hydroxypropranolol contains two hydroxy groups, which are both potential reaction sites. In order to assess the regioselectivity of 4-hydroxypropranolol glucuronidation, 4-methoxypropranolol was tested as an additional substrate because its aromatic hydroxy group is blocked by a methyl group. Thus, analysis of the glucuronidation of this substrate by a UGT allows the relevant reaction site of 4-hydroxypropranolol for the same enzyme to be inferred. 4-Methoxypropranolol was synthesized chemically according to a method adapted from Oatis et al. and Harps et al. [33,34] (Supplementary Material). The synthesis was stopped before the final step of demethylation, and thus, 4-methoxypropranolol was obtained. 

**Figure 5:**
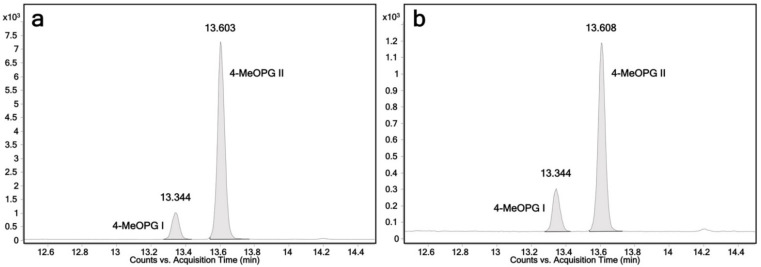
Chromatograms of 4-methoxypropranolol glucuronide I and 4-methoxypropranolol glucuronide II produced by UGT1A9 (**a**) and UGT2A1 (**b**). The displayed ion transition is *m*/*z* 466→116; 4-MeOPG I: 4-methoxypropranolol glucuronide I, retention time 13.3 min; 4-MeOPG II: 4-methoxypropranolol glucuronide II, retention time 13.6 min.

Due to their capability to glucuronidate 4-hydroxypropranolol, UGT1A7, UGT1A8, UGT1A9 and UGT2A1 were tested with 4-methoxypropranolol as a substrate. UGT1A9 and 2A1 were found to glucuronidate 4-methoxypropranolol, producing two glucuronides, while UGT1A7 and UGT1A8 were inactive. LC-MS chromatograms are shown in Figure 5a,b. Because of the lack of enantiopure (*R*)- or (*S*)-4-methoxypropranolol, the configuration of the two 4-methoxypropranolol glucuronide diastereomers cannot be assigned. Thus, they were labeled as glucuronide I and II. 4-Methoxypropranolol glucuronide I was eluted at 13.3 min, and 4-methoxypropranolol glucuronide II was eluted at 13.6 min. It may be hypothesized from the comparison of the elution orders of the propranolol enantiomers that the earlier eluting 4-methoxypropranolol glucuronide I corresponds to the *(R)*-enantiomer, while II may correspond to the *(S)* form. However, further evidence will be needed for confirmation.

### 2.4. Comparative Mechanistic Modeling for Human UGTs

**Figure 6:**
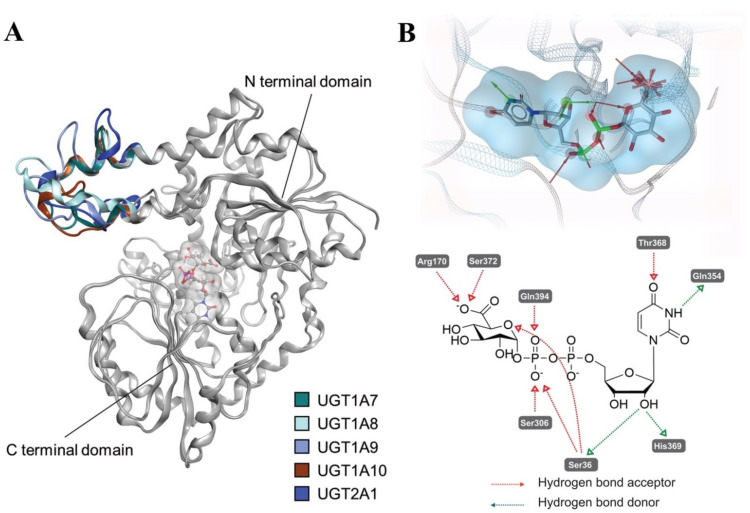
Comparison of the UGT1A7, UGT1A8, UGT1A9, UGT1A10 and UGT2A1 homology models. (**A**) Alignment of UGT1A7, UGT1A8, UGT1A9, UGT1A10 and UGT2A1 homology models. The major body was colored gray, and the most various part was colored for comparison: UGT1A7 (dark green), UGT1A8 (light green), UGT1A9 (light blue), UGT1A10 (brown) and UGT2A1 (dark blue). (**B**) Binding mode of UDPGA at the UGT1A7 binding site. The ligand molecular surface is presented transparently blue. 3D pharmacophores were generated to show the interactions between UDPGA and UGT1A7: hydrogen bond donors (HBD, green arrows), hydrogen bond acceptors (HBA, red arrows) and negative charge (red star). A 2D schematic diagram is shown below.

Human UGTs contain two major functional domains: A C-terminal domain (CTD) and an N-terminal domain (NTD), taking responsibility for cofactor binding and substrate recognition, respectively [35]. Due to the difficulty of crystallizing the membrane-bound UGTs, so far, only the structures of the CTDs of human UGT2B7 [36] and human UGT2B15 [37] have been experimentally solved (PDB entries 2O6L and 6IPB). In order to rationalize possible binding modes of propranolol and its derivatives in human UGT1A7, UGT1A8, UGT1A9, UGT1A10 and UGT2A1, homology models of these human UGTs were built (Figure 6). Superposed conformations of the UGTs homology models are shown in Figure 6A. The average root-mean-square deviations (RMSD) of the backbone Cα atoms from the optimized UGT1A7, UGT1A8, UGT1A9, UGT1A10 and UGT2A1 homology models (Figure 6A) compared with their template (PDB entry: 5GL5) are 1.13 Å, 1.21 Å, 1.18 Å, 1.48 Å and 1.48 Å, respectively. It is worth noting that the NTD loops (colored blue in Figure 6A) for the studied UGTs (residue number: 88–118 in UGT1A7 numbering) show a high degree of variability. This might influence the substrate recognition and catalytic activity while also contributing to the isoform selectivity.

**Figure 7:**
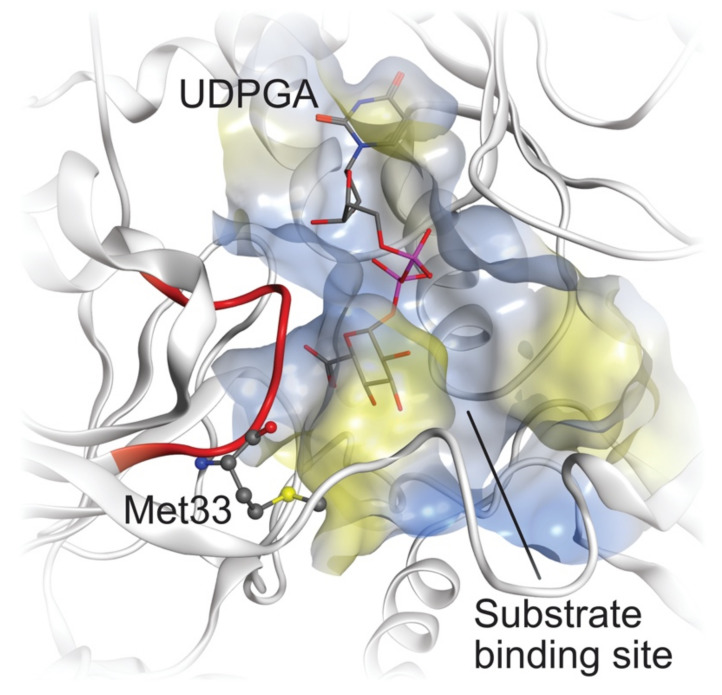
UGT1A7 binding site with receptor surface and cofactor (UDPGA) presented. The loop of residues 32–38 was colored red, and Met33 was shown in the ball and stick model.

UGT1A7, UGT1A8, UGT1A9 and UGT1A10 share high similarities with each other, while it is unexpected that UGT2A1 shows a similar activity with them but the other members of the UGT1A family do not. After alignment of our homology models and comparison of the sequence similarity of UGT1A7, UGT1A8, UGT1A9, UGT1A10 and UGT2A1 at their active site, we found that there is one loop (PM(D/E)GSHW, residue 32–38 in UGT1A7 numbering, colored red in Figure 7) at the substrate-binding site that is highly similar among all five isoforms involved in the glucuronidation of propranolol and 4-hydroxypropranolol. In this loop, Met33 is unique for these five isoforms compared with all the other isoforms in the UGT1 and UGT2 families. Direct interactions between residues in this loop and the substrates were observed in the docking results, suggesting that the loop with Met33 has a steric influence on the glucuronidation of propranolol and 4-hydroxypropranolol.

**Figure 8:**
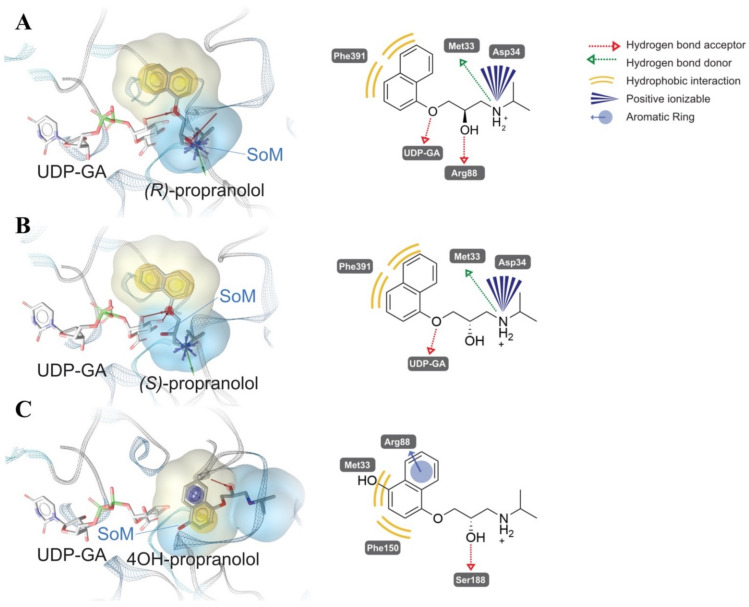
Suggested conformations of (*R*)-propranolol (**A**), (*S*)-propranolol (**B**) and 4-hydroxypropranolol (**C**) binding in the human UGT1A7 homology model. Three-dimensional pharmacophores were generated to present the interactions between propranolol and UGT1A7. SoM: Site of metabolism.

To rationalize the reported stereoselectivity of UGT1A7, UGT1A9, UGT1A10 and UGT2A1 on racemic propranolol, the docking experiments of (*S*)-propranolol, (*R*)-propranolol and 4-hydroxypropranolol were performed using the UGT1A7 homology model derived as described above. The enantiomers show different orientations fitting in the lipophilic interval between CTD and NTD besides UDPGA, as shown in Figure 8A,B.

For both (*S*)-propranolol and (R)-propranolol, the binding mode suggests lipophilic contacts between their naphthyl moiety and Phe391. Hydrogen bond interactions with UDPGA appeared in both (*S*)-propranolol and (*R*)-propranolol. Their amide groups are observed forming hydrogen bond interactions with Met33 and charge-charge interactions with Asp34 (Figure 8A,B). A comparison of the two docking conformations indicates that the interaction between the hydroxy group (site of metabolism) of (*R*)-propranolol and Arg88 in UGT1A7 might be the key contributor to the stereoselectivity observed in the biotransformation experiment. For (*S*)-propranolol, the hydroxy group is exposed freely towards the anomeric C atom of UDPGA, leading to a suitable position for the reaction to start. In contrast, the hydroxy group of (*R*)-propranolol interacts with Arg88, which forces the hydroxy group to be far from UDPGA. Similar interactions were also found in the conformations of UGT1A9 and UGT2A1 (Lys instead of Arg in a similar position). It is interesting to find that in UGT1A7 and UGT1A9, Asp87 attracts Arg88 to an orientation towards substrate by charge-charge interaction, thus interrupting a potential formation of α-helix as a stable secondary structure that exists in UGT1A10, in which the corresponding position of Asp87 is asparagine, a neutral residue.

Docking experiments suggest a different conformation of 4-hydroxypropranolol compared with propranolol, in which Met33 still plays a role in stabilizing the conformation (Figure 8C). In our model, both Met33 and Phe150 have hydrophobic contacts with the naphthyl moiety, and Ser188 forms a hydrogen bond with the hydroxy group. An additional cation-π interaction between 4-hydroxypropranolol and Arg88 exists. Thus, the overall orientation of 4-hydroxypropranolol at the substrate-binding site significantly differs from that of propranolol.

## 3. Discussion

Previous studies have already reported the glucuronidation of propranolol as such. Early research suggested that propranolol is metabolized via various pathways, including ring oxidation, side-chain oxidation and glucuronidation [38,39]. Glucuronidation may produce two glucuronide diastereomers due to the chirality of the substrate. It was found that the abundance of (*S*)-propranolol glucuronide exceeded four times that of the other diastereomer in both plasma and urine after the administration of racemic propranolol, suggesting that (*S*)-propranolol is the predominant substrate of glucuronidation in humans [24,26]. The stereoselective glucuronidation of propranolol was investigated before by enzyme kinetic analyses. In the case of UGT1A9 and UGT1A10, the K_m_ values for (*S*)-propranolol and (*R*)-propranolol differed much less than the corresponding V_max_ values, suggesting the major determinant may be the rate of glucuronic acid transfer to the already bound aglycone substrate [23].

Previous biotransformation studies with 15 recombinant human UGTs (1A1, 1A3–1A10, 2B4, 2B7, 2B10, 2B15, 2B17 and 2B28), all of which were expressed in baculovirus-infected insect cells, suggested that propranolol is mainly glucuronidated by UGT1A9, UGT1A10, UGT2B4 and UGT2B7 [23]. In this study, a complete reaction phenotyping with 19 enzymes of the UGT1 and UGT2 families on propranolol was performed. Besides UGT1A9 and UGT1A10, UGT1A7 and UGT2A1 were found to be capable of producing two propranolol glucuronides. However, no propranolol glucuronides produced by UGT2B4 and UGT2B7 were found in this study. If they were formed at all, their concentrations were lower than the detection limit. A potential reason could be the differences in the biotransformation system since recombinant fission yeast cells (*Schizosaccharomyces pombe*) were used in this study for the catalytic assays.

**Figure 9:**
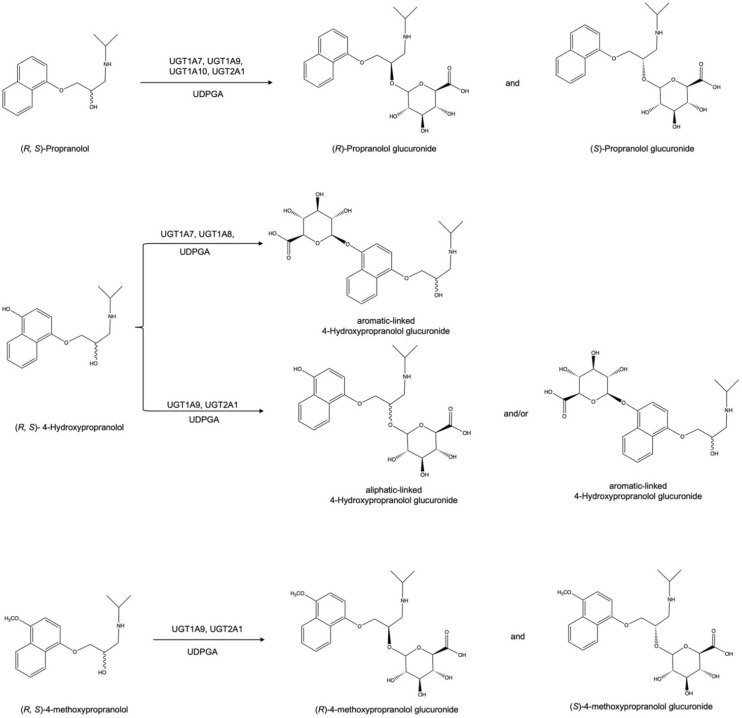
Reaction schemes for the glucuronidation of (*R*,*S*)-propranolol, (*R*,*S*)-4-hydroxypropranolol and (*R*,*S*)-4-methoxypropranolol.

Next, (*R*)-propranolol was used as a substrate to differentiate the two diastereomeric glucuronides (Figure 2). According to our biotransformation results, the relative biocatalytic yields of (*R*)-propranolol glucuronide were 31.5%, 23.9% and 48.9% of (*S*)-propranolol glucuronide for UGT1A7, UGT1A9 and UGT2A1, suggesting (*S*)-propranolol is the preferred substrate for the glucuronidation of these enzymes (Figure 1a,b,d). In contrast, UGT1A10 showed reverse stereoselectivity, with a relative yield of 21.2% for (*S*)-propranolol glucuronide compared to (*R*)-propranolol glucuronide (Figure 1c). A reaction scheme for the glucuronidation of (*R*,*S*)-propranolol, (*R*,*S*)-4-hydroxypropranolol and (*R*,*S*)-4-methoxypropranolol is shown in Figure 9.

To rationalize the observed stereoselectivity of UGT1A7, UGT1A9, UGT1A10 and UGT2A1 on racemic propranolol, the docking experiments of (*S*)-propranolol and (*R*)-propranolol were conducted using a UGT1A7 homology model derived as described in Section 4. The interaction between the hydroxy group (site of metabolism) of (*R*)-propranolol and Arg88 in UGT1A7 might be the key contributor to the stereoselectivity difference observed in the biotransformation experiments (Figure 8A,B). In the case of (*R*)-propranolol, Asp87 attracts Arg88 towards the hydroxy group by charge-charge interaction, resulting in the interaction between Arg88 and the hydroxy group. This forces the hydroxy group to be far away from the cofactor, UDPGA, thus hindering the transfer of glucuronic acid from the cofactor to the hydroxy group. For (*S*)-propranolol, because of its different orientation of binding with the enzyme, the same interference on the hydroxy group does not exist. The hydroxy group is free to be exposed towards the anomeric C atom of UDPGA, leading to a suitable situation for initiating the catalysis.

According to our biotransformation results, UGT1A7, UGT1A9 and UGT2A1 have the same selectivity towards (*S*)-propranolol. However, in the case of UGT1A10, (*R*)-propranolol is the preferred substrate. In UGT1A7 and UGT1A9, Asp87 contributes to the interaction between Arg88 and the hydroxy group on (*R*)-propranolol. Although this Arg does not exist in UGT2A1, a Lys at a similar position may also serve as a hydrogen bond donor for “suppressing” the interaction. However, in UGT1A10, the corresponding side chain of Asp87 is an asparagine. This neutral residue is not able to form charge-charge interactions with Arg88 like Asp87 in UGT1A7 and UGT1A9. Thus, the glucuronidation of (*R*)-propranolol by UGT1A10 is not affected.

4-Hydroxypropranolol has been demonstrated to be the major phase I metabolite of propranolol after oral administration and was found to have a similar beta-blocker effect as propranolol [40,41]. However, unlike propranolol, only limited information about the metabolism and elimination of 4-hydroxypropranolol is available. Here, we demonstrate that UGT1A7, UGT1A8, UGT1A9 and UGT2A1 are able to glucuronidate this compound (Figure 4).

While UGT1A8 was found to not glucuronidate propranolol and UGT1A10 showed no glucuronidation of 4-hydroxypropranolol, UGT1A7 and UGT1A9 showed similar results in the biotransformation of both substrates. The high similarity of their amino acid sequences contributes to their comparable preference for the substrates. However, the differences in the amino acid sequence of UGT2A1 compared with those four UGT1A isoenzymes is higher than the differences within the UGT1A subfamily at the cofactor binding site. After aligning and superposing the binding site of these four enzymes, a loop attracted our attention due to its high similarity. Therefore, we compared the sequences of all 19 UGTs and found that UGT1A7, UGT1A8, UGT1A9, UGT1A10 and UGT2A1 shared a highly similar protein sequence in this loop (PMDGSH for UGT1A7, UGT1A8, UGT1A9, UGT1A10 and PMEGSH for UGT2A1) as shown in Figure 7. The structures of glutamic acid (E) and aspartic acid (D) are highly similar, so their potential function in this loop was surmised to be identical. Comparing this loop in other UGT isoforms, Met33 was only present in UGT1A7, UGT1A8, UGT1A9, UGT1A10 and UGT2A1. Since direct interactions between Met33 and substrates were observed in docking results (Figure 8), that leads us to a hypothesis that the loop with Met33 has an influence on the glucuronidation of propranolol and 4-hydroxypropranolol.

It is worth noting that 4-hydroxypropranolol has two hydroxy groups, the aromatic hydroxy group on the naphthol ring and the aliphatic hydroxy group on the side chain. Both hydroxy groups can theoretically be glucuronidated by UGTs. It is difficult to determine the SoM by tandem MS because of the dominant cleavage at the glucuronidation position (Figure 3B). Derivatization of the substrate was, therefore, considered to be a helpful alternative. Some previous studies, where GC/MS analyses were performed after derivatization with hexamethyldisilane and diazomethane, suggested that the 4-hydroxypropranolol glucuronide collected from dog urine samples or incubation of microsomes is aromatic-linked [28,41]. However, the choice of derivatization reagents is critical because the secondary amine group of 4-hydroxypropranolol could be methylated before the aliphatic hydroxy group during the derivatization. Furthermore, the interpretation of the derivatized glucuronides is complicated, and sometimes, multiple MS is needed. In the case of 4-hydroxypropranolol glucuronide derivatized by 1,2-dimethylimidazole-4-sulfonyl chloride (DMISC), MS^3^ and MS^4^ analysis were performed to distinguish the aromatic-linked and aliphatic-linked glucuronides [42].

Theoretically, under the same separating condition, there should be two diastereomeric glucuronides if the glucuronidation position of 4-hydroxypropranolol is on the aliphatic hydroxy group such as propranolol. In our study, no hydroxy glucuronic diastereomers were found, and no stereoselectivity was detected. The potential reason could be the glucuronidation position is in the aromatic hydroxy group. The aromatic-linked glucuronic isomers are not separated under the analytical conditions employed. Therefore, in this study, 4-methoxypropranolol was used as a substrate (instead of derivatization of glucuronides) to determine the glucuronic position. Because the naphthol hydroxy group was blocked, no aromatic-linked glucuronides could be produced. Biotransformation with 4-methoxypropranolol showed that UGT1A9 and UGT2A1 were active and yielded two glucuronic diastereomers (Figure 5a,b), suggesting that those two UGTs also catalyzed aliphatic-linked glucuronidation of 4-hydroxypropranolol. UGT1A7 and UGT1A8 are inactive with 4-methoxypropranolol as a substrate, suggesting that they only catalyze aromatic-linked glucuronidation.

As mentioned in Section 2.4, the binding conformation of propranolol and 4-hydroxypropranolol is different because of additional interactions with the aromatic group (Figure 8C). Further docking experiments of UGT1A9 and UGT2A1 indicate there is no additional interaction between the methoxy group and the enzyme, suggesting that the conformation of UGT1A9 and UGT2A1 with propranolol and 4-methoxypropranolol are equivalent. Therefore, it is concluded that UGT1A9 and UGT2A1 prefer to glucuronidate the aromatic hydroxy group of 4-hydroxypropranolol. However, when the aromatic hydroxy group is blocked, they may also glucuronidate the aliphatic hydroxy group instead. In that case, their binding conformation with the enzyme is the same as propranolol. Comparing the situation with propranolol, we can suppose that 4-MeOPG I is the (*R*)-4-methoxypropranolol glucuronide and 4-MeOPG II is the (*S*)-isomer, which is in line with the hypothesis based on elution orders (Section 2.3). Further confirmation with enantiopure 4-methoxypropranolol as a substrate should be conducted in the future.

Certainly, there is a possibility that the mixture of two diastereomeric aliphatic-linked 4-hydroxypropranolol glucuronides and two aromatic-linked diastereomers were not separated under the conditions employed here. Those inferences about the preferences in the aromatic hydroxy group of UGT1A9 and UGT2A1 cannot prove that 4-hydroxypropranolol was solely converted to its aromatic-linked glucuronide. Furthermore, a potential reason for the multi-function of UGT1A9 and UGT2A1 in producing different products is that UGT1A9 and UGT2A1 show higher flexibility than other UGTs, as suggested by homology modeling and MD simulation results. The movement of the enzymes will result in a shift of the relative position of substrate and cofactor, thus satisfying the distance requirement for both aromatic and aliphatic glucuronidation.

## 4. Materials and Methods

### 4.1. Chemical and Reagents

Tris, KCl and Triton X-100 are from Carl Roth GmbH + Co. KG (Karlsruhe, Germany); UDPGA, (±)-propranolol, (*R*)-propranolol and (±)-4-hydroxypropranolol are from Sigma-Aldrich (St. Louis, Mo, USA); 4-methoxy-1-naphthol and epichlorohydrin are from ThermoFisher GmbH (Kandel, Germany); methylethylketone is from T-E-Klebetechnik (Hannover, Germany); potassium carbonate and NH_4_HCO_3_ are from VWR International GmbH (Darmstadt, Germany); isopropylamine is from TCI Europe N.V (Haven, Belgium); N-methyl-N-(trimethylsilyl)trifluoracetamid (MSTFA) is from Chemische Fabrik Karl Bucher GmbH (Waldstetten, Germany).

### 4.2. Synthesis of 4-Methoxypropranolol

The synthesis method of 4-methoxypropranolol was adapted from the method that Oatis et al. and Harps et al. applied for the synthesis of 4-hydroxypropranolol [33,34]. 4-Methoxy-1-naphthol (1 g) and potassium carbonate (2.5 g) were suspended in 12.5 mL methylethylketone (MEK) and 15 mL epichlorohydrin. The mixture was refluxed for 6 h in an oil bath. Then, the reaction mixture was filtrated to get rid of the potassium carbonate, and the solvent was evaporated. To the residue, 10 mL of isopropylamine and 1 mL of water were added, and the reaction mixture was refluxed for 5 h. After evaporation of the solvent, 50 mL hexane were added for recrystallization. The final product was cooled down to 4 °C overnight. The crystals were filtrated and dried, and redissolved in methanol to get the stock solution of 4-methoxypropranolol. The product was derivatized with MSTFA (N-methyl-N-(trimethylsilyl)trifluoracetamid) and analyzed by GC-MS for confirmation of the structure and purity.

### 4.3. GC-MS Analysis of 4-Methoxypropranolol

Analysis was performed on an Agilent 7890A gas chromatographic system (Agilent Technologies GmbH, Waldbronn, Germany) coupled to an Agilent 5975 C inert mass selective detector, equipped with an Agilent HP1 column (17 m, 0.2 mm, 0.11 μm) with helium as the carrier gas. The following parameters were used for the analysis of products: oven program; 150 °C, +10 °C/min to 250 °C (rate 1), +40 °C/min to 310 °C (rate 2), hold for 2 min, injection volume; 2 μL, split 16:1, injection temperature; 300 °C, electron ionization (EI): 70 eV, full scan mode from *m*/*z* 40 to *m*/*z* 1000. Prior to GC–MS analysis, the dried residues were derivatized with MSTFA to a final concentration of 100 ppm at 75 °C for 20 min. The GC-MS results are shown in Supplementary Materials.

### 4.4. Biotransformation with UGT Enzyme Bags

Enzyme bags for UGTs were obtained as described previously [12,14,43] with slight modifications. Fission yeast cells were first incubated on EMM solid medium with leucine at 30 °C for 3 days. Then the cells were incubated in 10 mL EMM liquid medium with leucine at 30 °C agitating at 230 rpm for 24 h. For each sample, a volume that corresponds to 5 × 10^7^ cells was pipetted to 1.5 mL Eppendorf tubes. Then, the cells were centrifuged at 4500 rpm for 5 min, and the supernatant was discarded. For permeabilization, 1 mL 0.3% Triton X-100 in Tris-KCL buffer was added to each sample, and the samples were incubated at 30 °C for 1 h at 150 rpm. After permeabilization, the cell pellets were washed with 1 mL NH_4_HCO_3_ buffer (50 mM, pH 7.8) three times to wash away the remaining detergent. After washing, the cell pellets, known as enzyme bags, were used for UGT-dependent reactions. Enzyme bags were resuspended in 200 μL NH_4_CO_3_ buffer (50 mM, pH 7.8) containing 1 mM UDPGA and 1 mM substrate and incubated for 15 h at 37 °C, 300 rpm in an incubator. Afterward, the mixtures were centrifuged at 4500 rpm for 10 min. The supernatants were taken for analysis by LC-MS/MS.

### 4.5. LC-MS/MS Analysis of Propranolol Glucuronides, 4-Hydroxypropranolol Glucuronides and 4-Methoxypropranolol Glucuronides

Analysis was performed on an Agilent Technologies 1290 infinity high-performance liquid chromatograph equipped with an Agilent 6495 Triple Quadrupole mass spectrometer (Agilent Technologies, Waldbronn, Germany). Propranolol, propranolol glucuronide, 4-hydroxypropranolol, 4-hydroxypropranolol glucuronide, 4-methoxypropranolol and 4-methoxypropranolol glucuronide were separated on an Agilent Poroshell Phenyl Hexyl column (100 mm × 3.0 mm, 1.9 μm).

The flow rate was 0.4 mL/min, and the column temperature was 25 °C. For the simultaneous detection of the substrate and glucuronides, the initial mobile phase composition was 98% water with 0.1% formic acid and 10 mM ammonium formate (A) and 2% acetonitrile with 0.1% formic acid, 10% water and 10 mM ammonium formate (B). The amount of A was decreased to 85% within 7 min, to 60% within 7–14 min and 5% within 14–15 min. Then, A maintained at 5% till the end, 17 min. The details of the transitions of analytes are listed in Table 2. The presented peak areas were provided by transitions of the highest intensity.

For the confirmation of glucuronic products, accurate mass tandem MS was recorded on an Agilent 6550 QTOF mass spectrometer (Agilent Technologies, Santa Clara, CA). MS parameters were set using positive ion mode with spectra acquired over a mass range of *m*/*z* 100–1000; capillary voltage 3500 V; gas temperature 200 °C; dry gas flow 12 L/min; collision energy: 30.3 eV for propranolol glucuronide, 31.2 eV for 4-hydroxypropranolol glucuronide.

### 4.6. Method Performance Characteristics

Stock solutions of the substrates were prepared at 2 mg/mL and stored at −20 °C until further use. The stock solution of propranolol was diluted by methanol to obtain 1, 2, 10, 20, 100, 200, 1000 and 2000 ng/mL standard solutions for calibration and the determination of the limit of detection (LOD). Linearity of the calibration was found in the range of 10–200 ng/mL with regression type proven by the Mandel test. Using a signal-to noise ratio of 3, the lowest concentration level (1 ng/mL) was found as LOD. The lower limit of quantitation (LOQ) was estimated below 10 ng/mL by a relative standard deviation below 20% at this level. Because of the lack of glucuronide reference substances, proper quantitation of their amounts in the samples is impossible for now.

However, for method performance evaluation with respect to the glucuronides, the LOQ was determined by analyzing a series of diluted incubation samples as a surrogate. A 1:50 (*v*:*v*) dilution of a pooled set of UGT1A9 incubations with blank samples still resulted in a relative standard deviation below 20%. Thus, it can be considered as LOQ. As the S/N ratio of the detected peaks for this dilution is >>3, the LOD is estimated as much lower.

The method precision for the glucuronides was evaluated by analyzing non-diluted incubation duplicates as high-concentration quality control (HQC), 0.2× and 0.125× diluted samples as two different medium concentration quality controls (MQC1 and MQC2), and 0.02× as low concentration quality controls (LQC). Mean peak areas with SD and the percentage of coefficient variation for (*R*)-propranolol glucuronide and (*S*)-propranolol glucuronide are reported in Table 3. The results of CV were generally within the acceptance values (below 15%), indicating that the analyte in the enzyme bags incubations could be determined with reliable precision. For 0.02× diluted samples, the CVs are around 20%, which is forgivable in this case because of the biological background and potential low concentration.

The matrix effects were evaluated by measuring the UGT1A9 incubation samples diluted with neat solvent (0.1×, 0.125×, 0.2× and 0.5×). The responses of the quantifier transitions were compared to determine whether a linear increase in the signal was obtained with differently diluted samples. Hence, the 10%, 12.5%, 20%, and 50% “matrix samples” were compared with the undiluted samples. The response of the 10% matrix sample should equal the 100% matrix sample when multiplied by 10 (analogously for the other dilutions). Relative standard deviations < 20% were realized in all these samples after back-calculation, demonstrating an irrelevant matrix effect.

### 4.7. Homology Modeling of UGT1A7, UGT1A8, UGT1A9, UGT1A10 and UGT2A1

Homology modeling was conducted on the SWISSMODEL server [44], with an input of the human UGT1A7, UGT1A8, UGT1A9, UGT1A10 and UGT2A1 sequence (UniProt, Identifiers: Q9HAW7, Q9HAW9, O60656, Q9HAW8 and P0DTE4), respectively. The crystal structure of sterol 3-beta-glucosyltransferase in complex with its cofactor UDP-glucose (PDB code: 5GL5 [45]) was chosen as the template, for all the homology modeling, since it has the most identical sequence with UGT2A1 (identity: 16.34, similarity: 29.6), second-most identical sequence with UGT1A7 (identity: 17.6%, similarity: 33.3%), UGT1A8 (identity: 16.83%, similarity: 33.2%), UGT1A9 (identity: 16.3%, similarity: 31.5%), UGT1A10 (identity: 17.1%, similarity: 32.7%) and the most similar co-crystallized ligand, UDP-glucose, compared with UDPGA. Structure refinement of the raw homology models was conducted in the MOE software package (Molecular Operating Environment 2020. 0901; Chemical Computing Group ULC, Montreal, QC, Canada). Protonate 3D embedded in MOE was used for protein protonation. Dihedral angle outliers in each model were corrected using the local energy minimizations feature of the MOE Protein Builder and the MOE loop modeler tool within the OPLS-AA force field [46]. UDPGA, the cofactor, was docked into the newly created models using GOLD v. 5.8.1 (Genetic Optimization for Ligand Docking, CCDC Software, Cambridge, UK) [47] with general steps as described in the molecular docking section below.

All-atom molecular dynamics (MD) simulations were performed with the selected docking pose of UDPGA in complex with the UGT1A7 homology model as a starting point. The complexes were prepared in MOE, and the environment systems were prepared in Maestro v. 11.7 (Schrödinger Release 2020-4: Maestro, Schrödinger, LLC., New York, NY, USA). Termini were capped automatically in Maestro. The enzyme complexes were solvated in a 15 Å padding box with TIP3P water model [48]. Sodium ions were added to the environment system for electric neutrality, and an additional 0.15 M of sodium chloride ions were added to simulate a physiological environment. A default seven-step protocol for system relaxation and equilibration was implemented. The temperature and pressure for the simulation were set at 300 K and 1.01325 bar, respectively. The prepared MD simulation system was carried out with the Desmond v. 5.5 [49] on water-cooled Nvidia RTX 2080 Ti graphical processing units (GPU) for 100 ns in five replicas using the OPLS-AA force field, with a recorded interval of 100 ps. The trajectories were wrapped and aligned in VMD v. 1.9.3 [50]. Coordinates of the complexes were written out in VMD. UDPGA conformation of a representative complex with a UDP1A7 homology model was written out and adapted to the original homology models. The new conformations of UDPGA in complexes with different human UGTs were energy-minimized in MMFF94 force field [51], respectively.

### 4.8. Molecular Docking

Docking experiments for ligands or cofactors in their respective models were carried out using GOLD v. 5.8.1. Search efficiency was set to 200%, and the genetic algorithm (GA) was set to run 30 times for each ligand or the cofactor. The docking center of UDPGA was defined by the position of the corresponding anomeric C atom of UDP-glucose, the cofactor of the homology modeling templet (PDB entry: 5GL5) structure, and a sphere of 18 Å around it. The docking center of propranolol and its derivatives were chosen according to the position of the anomeric C atom of UDPGA, respectively. Docking output was evaluated by GoldScore P450 scoring function. The resulting docking poses were energy minimized by employing the MMFF94 force field implemented in LigandScout v. 4.4.3 (Inte:ligand, Vienna, Austria) [52], in which, subsequently, visual inspection and 3D pharmacophore modeling were performed. Docking conformations were mainly chosen based on the distance of propranolol to the anomeric C atom of UDPGA and reasonable geometry. The conformations were further selected according to the interaction numbers between substrates and proteins as indicated by 3D pharmacophores.

## 5. Conclusions

In summary, a complete reaction phenotyping with 19 enzymes of the UGT1 and UGT2 families on propranolol and 4-hydroxypropranolol was performed in this study. Four UGTs (UGT1A7, UGT1A9, UGT1A10 and UGT2A1) catalyzed the glucuronidation of racemic propranolol. UGT1A10 showed opposite stereoselectivity compared to UGT1A7, UGT1A9 and UGT2A1. The preference of two propranolol enantiomers was rationalized by homology modeling and molecular docking, and Arg88 was found to play an important role in the conformation of (*R*)-propranolol within the active site of the enzymes.

For the glucuronidation of 4-hydroxypropranolol, four UGTs (UGT1A7, UGT1A8, UGT1A9 and UGT2A1) were found to be active. UGT1A9 and UGT2A1 were able to glucuronidate 4-methoxypropranolol, while UGT1A7 and UGT1A8 did not. Therefore, it is hypothesized that both the aromatic hydroxy group and aliphatic hydroxy group of 4-hydroxypropranolol can be glucuronidated by different UGT isoforms. It is expected that those findings may contribute to the study of the stereoselectivity of enzymes from the geometry field in the future.

## Figures and Tables

**Table 1:** The activity of 19 human UGTs on (*R*)-propranolol, (*S*)-propranolol, 4-hydroxypropranolol and 4-methoxypropranolol.

UGTs	(*R*)-Propranolol	(*S*)-Propranolol	4-Hydroxypropranolol	4-Methoxypropranolol ^1^
UGT1A3	-	-	-	**
UGT1A4	-	-	-	**
UGT1A5	-	-	-	**
UGT1A6	-	-	-	**
UGT1A7	+ +	+ + +	+ + + +	-
UGT1A8	-	-	+ + + +	-
UGT1A9	+ + +	+ + + +	+ + + +	+ + + +
UGT1A10	+ +	+	-	-
UGT2A1	+ + +	+ + + +	+ + +	+ + + +
UGT2A2	-	-	-	**
UGT2A3	-	-	-	**
UGT2B4	-	-	-	**
UGT2B7	-	-	-	**
UGT2B10	-	-	-	**
UGT2B11	-	-	-	**
UGT2B15	-	-	-	**
UGT2B17	-	-	-	**
UGT2B28	-	-	-	**

“ + ” for peak area < 100; “ + + ” for peak area 100–500; “ + + + ” for peak area 500–2500; “ + + + +” for peak area > 2500, “**” The substrate was not tested by respective UGT. ^1^ The activity of UGT1A9 and UGT2A1 on 4-methoxypropranolol is determined by the peak area of 4-methoxypropranolol glucuronide II.

**Table 2:** Transitions for all analytes in MRM.

Analytes	Precursor Ions (*m*/*z*)	Product Ions (*m*/*z*)	CE (V)	ESI
Propranolol glucuronide	436	258	12	+
	436	183	16	+
	436	116	28	+
4-Hydroxypropranolol glucuronide	452	116	28	+
	452	72	44	+
4-Methoxypropranolol glucuronide	466	288	12	+
	466	116	28	+
	466	72	44	+

**Table 3:** Precision of (*R*)-propranolol glucuronide and (S)-propranolol glucuronide (n.d. represents the non-diluted incubation samples of propranolol with enzyme bags of isoform UGT1A9).

Analytes	Level	Precision	
		Mean Peak Area ± SD	CV (%)
(*R*)-Propranolol glucuronide	0.02×	31.19 ± 6.02	19.3
	0.125×	251.93 ± 20.08	7.9
	0.2×	385.59 ± 20.61	5.3
	n.d.	1329.77 ± 91.07	6.8
*(S)*-Propranolol glucuronide	0.02×	120.67 ± 24.60	20.4
	0.125×	883.12 ± 68.86	7.8
	0.2×	1362.62 ± 16.42	1.2
	n.d.	4880.75 ± 18.84	0.3

## Data Availability

Raw data are stored at the authors.

## Supplementary Materials

The following supporting information can be downloaded at: https://www.mdpi.com/article/10.3390/ijms23137476/s1.
