# Supplementary material for: Complete Reaction Phenotyping of Propranolol and 4-Hydroxypropranolol with the 19 Enzymes of the Human UGT1 and UGT2 Families
Source: Int J Mol Sci. 2022 Jul 5;23(13):7476. doi: 10.3390/ijms23137476 (PMC9267274; doi:10.3390/ijms23137476)
Supplement: Supplementary file 1 [file ijms-23-07476-s001.zip › ijms-1783623-supplementary.pdf]

Supplemental Materials

Synthesis of 4-methoxypropranolol

The synthesis of 4-methoxypropranolol was adapted from the methods described by Oaties et al. and Harps et al. [1,2] for the synthesis of 4-hydroxypropranolol. The reaction stopped before demethylation to keep the methyl group. The product was derivatized with MSTFA and analyzed by GC/MS for structure confirmation. The TMS-derivative of 4-methoxypropranolol was eluted at 8.27 min (Supplemental Figure 1a). The MS showed the molecular ion $M^{+\bullet}$ (m/z 361.3), as well as the characteristic fragment ions m/z 174.1 and m/z 72.1 (Supplemental Figure 1b).

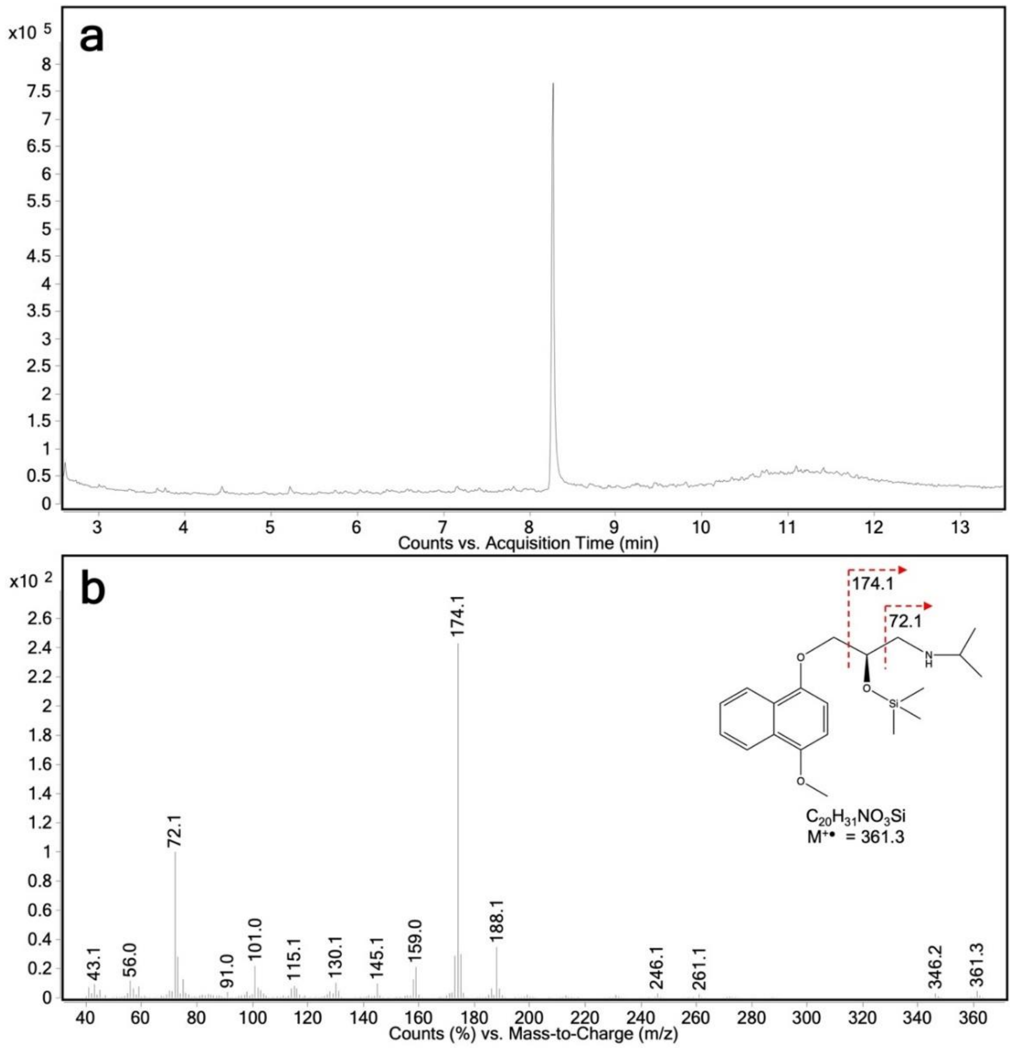

Figure S1. GC/MS analysis of O-TMS-derivative of 4-methoxypropranolol. (a) Total ion chromatogram, retention time 8.27 min; (b) MS spectrum, $M^{+\bullet}$ = 361.3

References

1. Oatis, J.E.; Russell, M.P.; Knapp, D.R.; Walle, T. Ring-hydroxylated propranolol: synthesis and .beta.-receptor antagonist and vasodilating activities of the seven isomers. *Journal of Medicinal Chemistry* **1981**, *24*, 309-314, doi:10.1021/jm00135a014.
2. Harps, L.C.; Schipperges, S.; Bredendiek, F.; Wuest, B.; Borowiak, A.; Parr, M.K. Two dimensional chromatography mass spectrometry: Quantitation of chiral shifts in metabolism of propranolol in bioanalysis. *J Chromatogr A* **2020**, *1617*, 460828, doi:10.1016/j.chroma.2019.460828.
